# Supplementary material for: Pharmacokinetic Profiling Using 3H-Labeled Eggshell Membrane and Effects of Eggshell Membrane and Lysozyme Oral Supplementation on DSS-Induced Colitis and Human Gut Microbiota
Source: Int J Mol Sci. 2025 Sep 18;26(18):9102. doi: 10.3390/ijms26189102 (PMC12471195; doi:10.3390/ijms26189102)
Supplement: Supplementary file 1 [file ijms-26-09102-s001.zip › ijms-3804537_Supplementary Table S1 (R2).pdf]

Supplementary Table S1 Concise summary of the ARRIVE checklist for the mouse study

Item	Tracer study (^3H-ESM biodistribution)	DSS colitis model (ESM/LYZ supplementation)
**1. Study design**	Time-course tracer study; single oral ^3H-ESM; no control; unit = mouse	4 groups: Control, DSS-only, DSS+ESM, DSS+LYZ; unit = mouse
**2. Sample size**	n = 3–4 per time point (0.5–72 h); total 22	n = 4 per group; 2 experiments (3 d & 5 d DSS); total 32
**3. Inclusion/Exclusion**	No exclusions; all data included	No criteria; all animals included
**4. Randomisation**	Not applicable; homogeneous C57BL/6J used and all same treatment	Not used; homogeneous C57BL/6J used
**5. Blinding**	Not applicable; no group allocation	Not feasible; experimenter aware; cages labeled
**6. Outcome measures**	Radioactivity in serum, tissues, excreta (0.5–72 h)	Disease Activity Index (DAI: weight, stool, bleeding) at days 3 & 5
**7. Statistics**	t-test, one-way ANOVA, Tukey HSD; $p < 0.05$	t-test, one-way ANOVA, Tukey HSD; $p < 0.05$
**8. Animals**	Female C57BL/6J, 6 wks, healthy	Female C57BL/6J, 6 wks, healthy, no mortality
**9. Procedures**	A single oral dose of tritium-labeled eggshell membrane (5 mg [^{3}H]ESM, 1.3×10^4 Bq) suspended in MediGel	Oral ESM (7.4 mg/kg), LYZ (5.99 mg/kg), or MediGel only, once daily for 7 days; 2% DSS in water for all but control; sampling on days 3 and 5.
**10. Results**	^3H distribution quantified; descriptive pharmacokinetics; ANOVA significance	DAI increased with DSS; ESM/LYZ reduced severity; control DAI = 0
