# Supplementary material for: Pharmacokinetic Profiling Using 3H-Labeled Eggshell Membrane and Effects of Eggshell Membrane and Lysozyme Oral Supplementation on DSS-Induced Colitis and Human Gut Microbiota
Source: Int J Mol Sci. 2025 Sep 18;26(18):9102. doi: 10.3390/ijms26189102 (PMC12471195; doi:10.3390/ijms26189102)
Supplement: Supplementary file 1 [file ijms-26-09102-s001.zip › ijms-3804537_Supplementary Table S2 (R2).pdf]

Supplementary Table S2. Risk of Bias Assessment

Domain	Risk of Bias Judgment	Supporting Information
**Bias arising from the randomization process**	Low risk	Randomisation was performed using simple randomisation with a computer-generated allocation sequence prepared by an independent third party not involved in the study. No restrictions such as stratification or blocking were applied. Allocation was concealed using an ID-linked assignment table accessible only to the principal investigator. Participants were managed by ID codes to ensure allocation concealment, and group assignments remained undisclosed to all other research staff and participants.
**Bias due to deviations from intended interventions**	Low risk	A double-blind design was employed. Both participants and study personnel were unaware of group assignments throughout the trial. The placebo was visually and taste-wise indistinguishable from the ESM-based supplement. Gut microbiota analysis was performed by an independent external laboratory blinded to group allocation. Participant compliance and health condition were monitored using intake logs and structured diaries.
**Bias due to missing outcome data**	High risk	The intervention group had 3 dropouts (30%), exceeding the 20% threshold. Reasons included insufficient sample collection throughout the study (n = 2) and exclusion due to atypical gut microbiota profile (n = 1), potentially introducing attrition bias. No intention-to-treat analysis was performed.
**Bias in measurement of the outcome**	Low risk	Gut microbiota composition was analyzed using the Nagashima method (modified T-RFLP) by an independent external laboratory blinded to group allocation.
**Bias in selection of the reported result**	Some concerns	No a priori statistical analysis plan was pre-registered. Outcomes were analyzed and reported as collected. The sample size (n = 10 per group) was based on feasibility (recruitment, duration, funding), not a priori calculation; post-hoc power analyses and effect sizes were performed to assess statistical validity.
**Overall risk of bias**	Some concerns	Although randomization and blinding were appropriately conducted, the high dropout rate, absence of intention-to-treat analysis, and lack of pre-specified statistical plan increased the overall risk of bias.
