# Supplementary material for: Pharmacokinetic Profiling Using 3H-Labeled Eggshell Membrane and Effects of Eggshell Membrane and Lysozyme Oral Supplementation on DSS-Induced Colitis and Human Gut Microbiota
Source: Int J Mol Sci. 2025 Sep 18;26(18):9102. doi: 10.3390/ijms26189102 (PMC12471195; doi:10.3390/ijms26189102)
Supplement: Supplementary file 1 [file ijms-26-09102-s001.zip › ijms-3804537_Supplementary Table S4 (R2).pdf]

Supplementary Table S4. Baseline demographic characteristics of the study groups

	Control group (n= 10)	ESM group (n= 7)
Sex (Male: Female) (number)	4 : 6	2 : 5
Age (year)	39.1 ± 14.6	45.6 ± 16.1
Height (cm)	166.0 ± 5.4	163.1 ± 5.7
Baseline weight (kg)	61.3 ± 9.0	57.3 ± 7.0
Weight after 8 weeks (kg)	62.0 ± 9.0	57.2 ± 7.1
Baseline BMI (kg/m^2)	22.3 ± 3.1	21.6 ± 3.1
BMI after 8 weeks (kg/m^2)	22.4 ± 2.8	21.6 ± 3.1
