# Supplementary material for: Pharmacokinetic Profiling Using 3H-Labeled Eggshell Membrane and Effects of Eggshell Membrane and Lysozyme Oral Supplementation on DSS-Induced Colitis and Human Gut Microbiota
Source: Int J Mol Sci. 2025 Sep 18;26(18):9102. doi: 10.3390/ijms26189102 (PMC12471195; doi:10.3390/ijms26189102)
Supplement: Supplementary file 1 [file ijms-26-09102-s001.zip › ijms-3804537_Supplementary Table S5ab (R2).pdf]

Supplementary Table S5a. Results of temporal changes in gut microbiota α-diversity as measured by the Shan-non-Wiener diversity index (H).

	Control		ESM					
Weeks	average	SD	average	SD	p-value	statistically significant	d	Power
0	1.48	0.17	1.62	0.18	0.068		0.80	0.40
1	1.45	0.11	1.73	0.13	0.0001	***	2.32	0.99
2	1.48	0.16	1.70	0.16	0.008	**	1.38	0.74
3								
4	1.42	0.18	1.68	0.11	0.002	**	1.74	0.91
5								
6								
7								
8	1.39	0.16	1.78	0.21	0.0002	***	2.09	0.98

*p < 0.05, **p < 0.01, ***p < 0.001.

Supplementary Table S5b. Results of temporal changes in the proportion of beneficial bacteria

	Control		ESM					
Weeks	average	SD	average	SD	p-value	statistically significant	d	Power
0	11.58	5.62	10.48	5.96	0.352		0.19	0.07
1	13.00	8.90	15.17	4.83	0.283		0.30	0.09
2	12.30	3.46	13.41	9.07	0.364		0.16	0.06
3								
4	8.66	3.69	14.22	4.55	0.007	**	1.34	0.72
5								
6								
7								
8	9.18	4.40	16.61	10.76	0.03	*	0.90	0.40

*p < 0.05, **p < 0.01, ***p < 0.001.
