# Supplementary figures and images for: Pharmacokinetic Profiling Using 3H-Labeled Eggshell Membrane and Effects of Eggshell Membrane and Lysozyme Oral Supplementation on DSS-Induced Colitis and Human Gut Microbiota
Source: Int J Mol Sci. 2025 Sep 18;26(18):9102. doi: 10.3390/ijms26189102 (PMC12471195; doi:10.3390/ijms26189102)

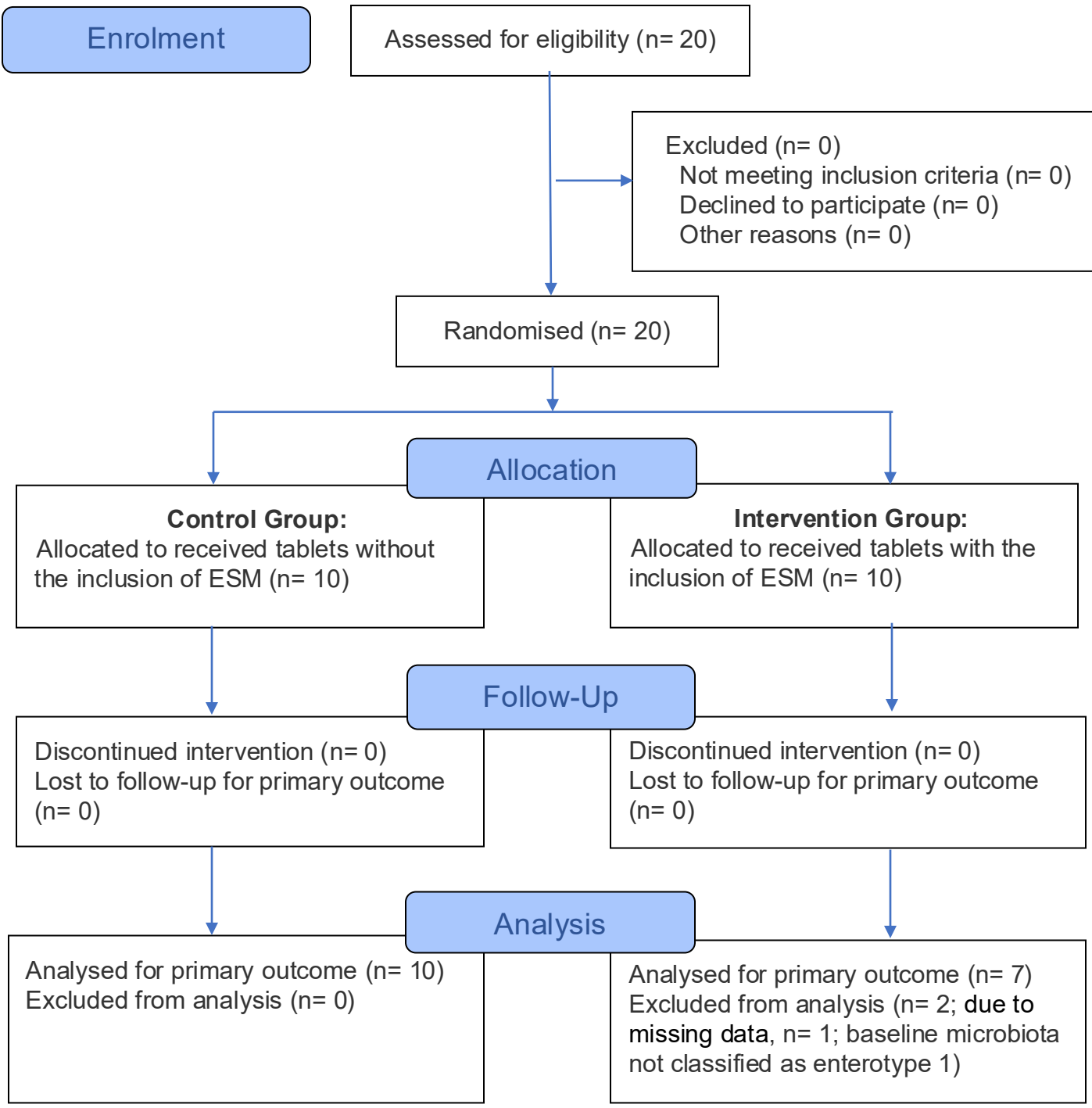

Supplementary Fig S1. Flow diagram of the study procedure.

Supplement: Supplementary file 1 [file ijms-26-09102-s001.zip › ijms-3804537_Supplementary Fig. S1.pdf]
